# Long noncoding RNA CRNDE promotes colorectal cancer cell proliferation via epigenetically silencing DUSP5/CDKN1A expression

**DOI:** 10.1038/cddis.2017.328

**Published:** 2017-08-10

**Authors:** Jie Ding, Juan Li, HaiYan Wang, Yun Tian, Min Xie, XueZhi He, Hao Ji, Zhonghua Ma, Bingqing Hui, Keming Wang, Guozhong Ji

**Affiliations:** 1 Department of Oncology, Second Affiliated Hospital, Nanjing Medical University, Nanjing, Jiangsu, People’s Republic of China; 2 Department of Ultrasonography, Second Affiliated Hospital, Nanjing Medical University, Nanjing, Jiangsu, People’s Republic of China; 3 Center for Reproduction and Genetics, Suzhou Municipal Hospital, Nanjing Medical University Affiliated Suzhou Hospital, Suzhou, Jiangsu, People’s Republic of China; 4 Department of Biochemistry and Molecular Biology, Nanjing Medical University, Nanjing, Jiangsu, People’s Republic of China; 5 Department of Digestive Endoscopy and Medical Center for Digestive Diseases, Second Affiliated Hospital, Nanjing Medical University, Nanjing, Jiangsu, People’s Republic of China

## Abstract

Evidence indicates that long non-coding RNAs (lncRNAs) play a critical role in the regulation of tumor cellular processes, such as proliferation, apoptosis, and metastasis. LncRNA CRNDE (Colorectal Neoplasia Differentially Expressed) is located at human chromosome 16 and has been found overexpressed in a variety of cancers including colorectal cancer (CRC). In this paper, we report that lncRNA CRNDE expression was remarkably upregulated in CRC tissues and that lncRNA CRNDE overexpression was positively correlated with advanced pathological stages and larger tumor sizes. In addition, the knockdown of CRNDE significantly suppressed proliferation and caused apoptosis of CRC cells both *in vitro* and *in vivo*. Furthermore, RNA immunoprecipitation and chromatin immunoprecipitation assays demonstrated that lncRNA CRNDE could epigenetically suppress the expressions of dual-specificity phosphatase 5 (DUSP5) and CDKN1A by binding to EZH2 (the key components of Polycomb repressive complex 2 (PRC2)), thus promoting CRC development. In conclusion, our data suggest that the lncRNA CRNDE promotes the progression of CRC and is a potential therapeutic target for CRC intervention.

Although considerable headway has been achieved in the diagnosis and therapy of colorectal cancer (CRC) in the past decade, it still causes significant morbidity and mortality, especially in the developed world.^1, 2, 3^ According to the latest cancer statistics, CRC is the third most common cancer and third leading cause of cancer death worldwide.^2^ Unfortunately, a great majority of the patients are diagnosed at an advanced stage that has passed the optimal time for a radical operation.^4^ As is known to all, CRC carcinogenesis is a complicated biological process involving multiple genomic variation and biological processes.^5^ Therefore, further study of the molecular mechanisms involved in the development and progression of CRC is essential to gain the targeted strategies to ease the burden of the disease.

With the development of whole-genome sequencing technology, it has gradually been revealed that protein-coding genes constitute only small portions (∼2%) of the genome encode proteins, whereas the vast majority of the human genome is transcribed into noncoding RNAs including microRNA genes, long noncoding RNA (lncRNA) genes, and pseudogenes.^6, 7^ Recently, lncRNAs as the new class of noncoding RNA molecules have become a hot topic because of their abnormal expression in human diseases, especially in tumor.^8, 9, 10, 11^ The role of lncRNAs in tumorigenesis and development has emerged from the water and been identified as the future diagnostic, therapeutic, or prognostic cancer biomarkers, including for CRC.^12, 13, 14^ For instance, lncRNA-linc00152 downregulated by miR-376c-3p restricts viability and promotes apoptosis of colorectal cancer cells;^15^ a high expression of lncRNA-NEAT1 (nuclear paraspeckle assembly transcript 1) predicted an unfavorable prognosis in patients with digestive system malignancies.^16^ Our previous studies also demonstrated that lncRNA-HOXA-AS2 (HOXA cluster antisense RNA 2) represses CDKN1A and KLF2 expression transcription by binding with EZH2 (enhancer of zeste homolog 2) and LSD1 in CRC;^17^ lncRNA-LOC554202 could induce apoptosis via the caspase cleavage cascades in CRC cells;^18^ lncRNA-HOTTIP (HOXA transcript at the distal tip) promotes colorectal cancer growth partially via silencing of CDKN1A expression.^19^

LncRNA CRNDE (Colorectal Neoplasia Differentially Expressed) is located at human chromosome 16 and has been found overexpressed in a variety of cancers including CRC.^20, 21, 22^ A large number of studies have shown that CRNDE plays an important role in the occurrence and development of CRC, indicating a poor prognosis.^23^ The upregulation of CRNDE expression has been shown to promote cell proliferation, migration, and invasion while inhibiting apoptosis of CRC.^24, 25^ Nevertheless, the underlying molecular mechanisms involved in CRNDE interactions are complex and diverse. It may function as a competing endogenous RNA to promote CRC metastasis and oxaliplatin resistance by sponging miR-136,^25^ or via miR-181a-5p-mediated regulation of Wnt/*β*-catenin signaling promotes colorectal cancer cell proliferation and chemoresistance.^24^ However, the above studies have singly pointed that CRNDE plays an oncogene function by regulating miRNA. The more effective and complex mechanisms of CRNDE deserve further study.

Given the importance of CRNDE in CRC, in the present study we investigated that CRNDE expression was remarkably upregulated in CRC tissues and that CRNDE overexpression was positively correlated with advanced pathological stages and larger tumor sizes. In addition, the knockdown of CRNDE significantly suppressed proliferation and caused apoptosis of CRC cells both *in vitro* and *in vivo*. Furthermore, RNA immunoprecipitation and chromatin immunoprecipitation assays demonstrated that lncRNA CRNDE could epigenetically suppress the expressions of DUSP5 (a negative regulator of mitogen-activated protein kinase (MAPK) signaling pathway) and CDKN1A by binding to EZH2 (the key components of Polycomb repressive complex 2 (PRC2)), thus promoting CRC development. In conclusion, our data suggest that the lncRNA CRNDE promotes the progression of CRC and is a potential therapeutic target for CRC intervention.

## Results

### CRNDE is upregulated in human CRC tissues and is positively correlated with larger tumor size, advanced TNM stage, and lymph node metastasis

**Figure 1:**
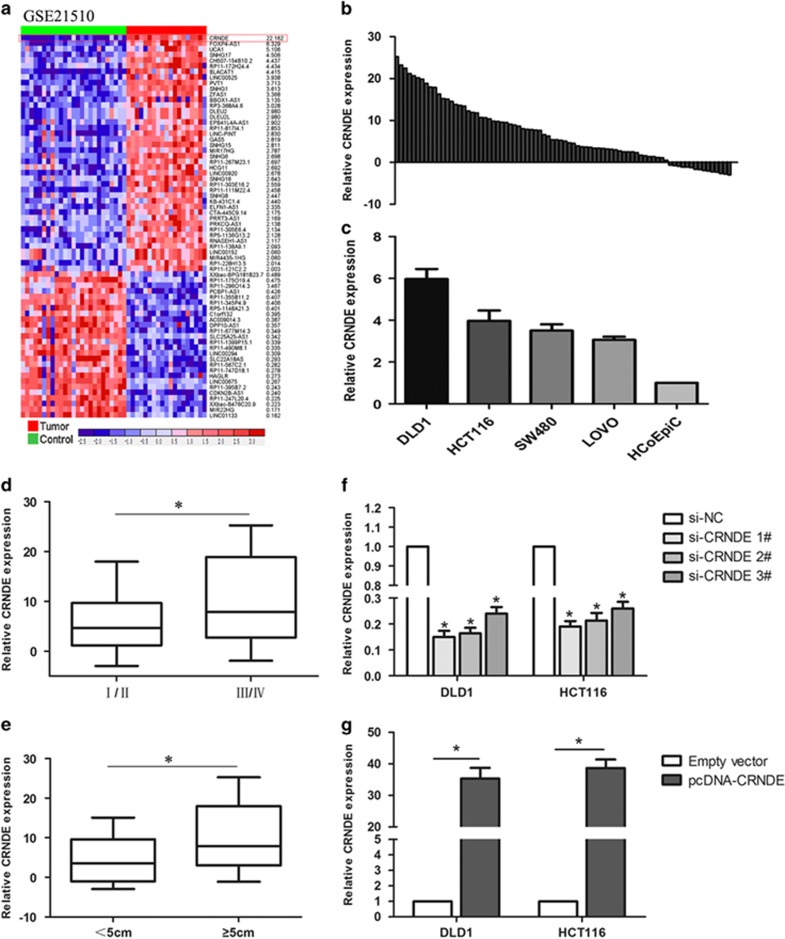
Relative expression of CRNDE in colorectal cancer tissues and cells compared with adjacent normal tissues and normal colonic epithelial cells. (**a**) Relative expression of CRNDE in human colorectal cancerous tissues compared with noncancerous tissue via GSE21510 data analysis. (**b**) The relative expression of CRNDE in colorectal cancer tissues (*n*=80) compared with corresponding nontumor tissues (*n*=80). CRNDE expression was examined by qRT-PCR and normalized to GAPDH expression. The results are presented as the fold change in tumor tissues relative to normal tissues (presented as −ΔΔCT). ΔCt value was determined by subtracting the GAPDH Ct value from the CRNDE Ct value. The −ΔΔCt value was calculated from the ΔCt value of the normal tissue control minus the ΔCt value of the CRC tissue. (**c**) CRNDE expression was assessed by qRT-PCR in colorectal cancer cell lines (DLD1, HCT116, SW480, and LOVO) and compared with the normal human colonic epithelial cell line (HCoEpiC). (**d** and **e**) The data are presented as the relative expression levels in tumor tissues. CRNDE expression was significantly increased in patients with a higher pathological stage and larger tumors. (**f** and **g**) qRT-PCR analysis of CRNDE expression levels following the treatment of DLD1 and HCT116 cells with si-CRNDE 1, 2, pcDNA-CRNDE, or the negative control. *P<0.05

We first analyzed the expression level of the CRNDE in human CRC tissues by using microarray data downloaded from the Gene Expression Omnibus (GEO; GSE21510), and found that the CRNDE expression level was significantly upregulated in CRC tissues compared with that in normal tissues (Figure 1a). The results of the CRC database downloaded from TCGA are consistent with the above findings (Supplementary Figure S1). Then, we examined CRNDE expression levels in 80 CRC tissue samples and matched adjacent normal tissues by performing quantitative real-time PCR (qRT-PCR) analysis. Similarly, this showed that CRNDE expression was significantly upregulated in CRC tissues compared with that in their normal counterparts (Figure 1b). Increased CRNDE expression was also validated in CRC cells, including DLD1, HCT116, SW480, and LOVO cells (*P*<0.05) (Figure 1c). To assess the significance of CRNDE expression in CRC, the clinicopathological features are summarized in Table 1. As shown in Figures 1d and e, upregulated CRNDE expression is closely related to increased tumor size and advanced TNM stage in the CRC patients. These findings imply that CRNDE may function as an oncogene involved in the tumorigenesis of CRC.

### SiRNA selection for CRNDE silencing or overexpression in CRC cells

To observe the functional relevance of CRNDE in CRC cells, we chose the DLD1 and HCT116 cell lines for further studies because they exhibited the highest expression levels compared with that in the normal colon epithelium cell line (HCoEpiC) (*P*<0.05) (Figure 1c). Next, we designed three different CRNDE short interfering RNAs (siRNAs) and one overexpression plasmid for transfection into the above two CRC cell lines. The qRT-PCR analysis was used to detect the transfection efficiency (Figures 1e and f). Of the three siRNAs, si-CRNDE 1 and 2 exhibited the better silencing capacity. Meanwhile, CRNDE expression was increased in all two CRC cell lines compared with the negative control following transfection with pcDNA-CRNDE. Thus, we selected si-CRNDE 1, 2, and pcDNA-CRNDE in all further experiments for CRNDE silencing or overexpressing, respectively.

### CRNDE promotes CRC cell proliferation *in vitro*

**Figure 2:**
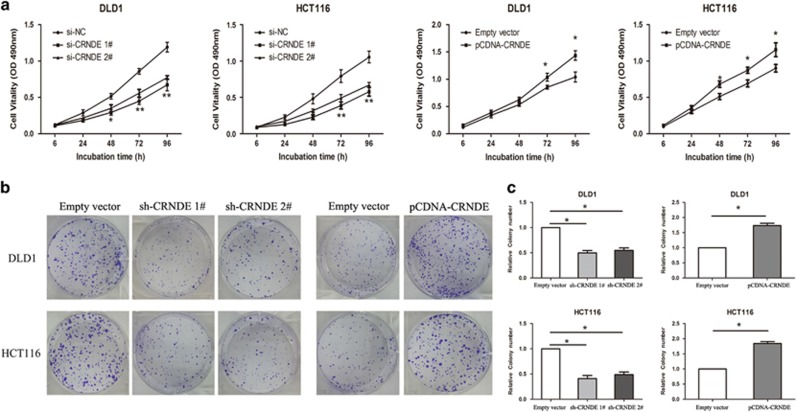
CRNDE promotes CRC cell proliferation *in vitro.* (**a**) A MTT assay was performed to determine the proliferation of DLD1 and HCT116 cells following treatment with si-CRNDE 1, 2, pcDNA-CRNDE, or the negative control. The data represent the mean±S.D. from three independent experiments. (**b**) Colony formation assays were performed to determine the proliferation of sh-CRNDE-transfected DLD1 and HCT116 cells or overexpression plasmid-transfected DLD1 and HCT116 cells. Experiments were performed in triplicate. (**c**) The colonies were counted and captured. Error bars indicate mean±S.D. *P<0.05 and **P<0.01

To further determine the potential biological role of CRNDE in CRC cell proliferation, we performed a MTT assay. The results showed that the growth vitality of DLD1 and HCT116 cells transfected with siRNA-CRNDE 1 or 2 was significantly inhibited compared with control groups. In contrast, CRNDE overexpression promoted the cell growth capacity (*P*<0.05, Figure 2a). The colony formation assays were performed to detect the tumorigenicity of CRC cells. The results suggest that the CRC cell colony numbers significantly decreased following knockdown of CRNDE but increased in CRC cells overexpressing CRNDE (*P*<0.05, Figures 2b and c). Taken together, these findings indicate that CRNDE can markedly promote the proliferation of CRC cells.

### Downregulation of CRNDE promotes G1 arrest and causes apoptosis in CRC cells *in vitro*

**Figure 3:**
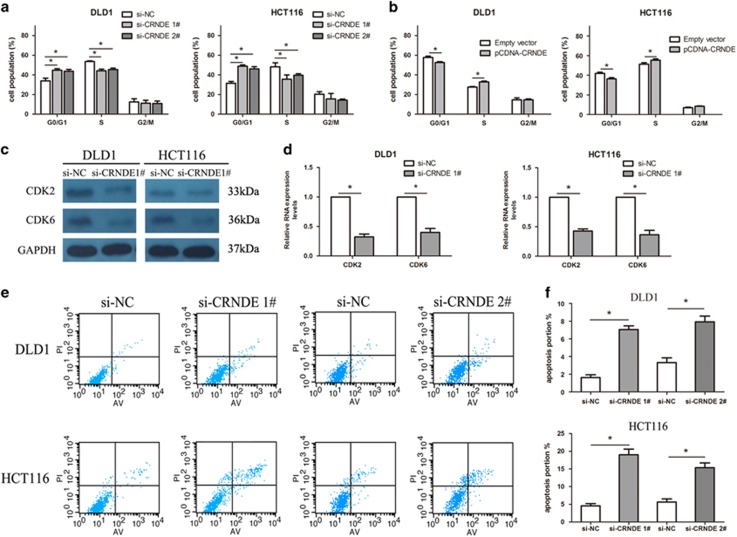
Downregulation of CRNDE promotes G1 arrest and causes apoptosis in CRC cells *in vitro.* (**a** and **b**) The bar chart represents the percentage of cells in G0/G1, S, or G2/M phase, as indicated. (**c** and **d**) Western blot analysis of CDK2 and CDK 6 after si-CRNDE 1 or si-NC transfection in DLD1 and HCT116 cells. GAPDH protein was used as an internal control. (**e** and **f**) The percentage of apoptotic cells was determined by flow cytometric analysis. The data represent the mean±S.D. from three independent experiments. *P<0.05

To assess whether the effects of CRNDE on CRC cell proliferation are the result of CRNDE-mediated changes in cell cycle progression, we examined cell cycling in CRC cells by flow cytometry. The results of si-CRNDE 1, 2, or si-NC transfection for 48 h showed that CRNDE knockdown increased the percentage of cells in G0/G1 phase and decreased the percentage of cells in S and G2/M phase compared with control cells (*P*<0.05) (Figure 3a). As shown in Figure 3b, overexpressing CRNDE promoted an obvious reduction in the G0/G1 phase, with a significant arrest in the number of cells in the S phase (*P*<0.05). Consistent with this finding, the results of western blot analysis showed that the protein levels of CDK2 (cyclin-dependent kinase 2) and CDK6 were significantly changed in si-CRNDE-treated cells (Figures 3c and d), confirming that CRNDE is involved in cell-cycle regulation. Moreover, we performed flow cytometric analysis to characterize the level of apoptosis of CRC cells that also reflect CRC cell growth vitality. The results demonstrated that the silencing of CRNDE expression significantly increased in CRC cell apoptosis (*P*<0.05, Figure 3e). These investigations suggest that CRNDE exerts critical influences on CRC cells by affecting both the cell cycle and apoptosis.

### Knockdown of CRNDE inhibits CRC tumorigenesis *in vivo*

**Figure 4:**
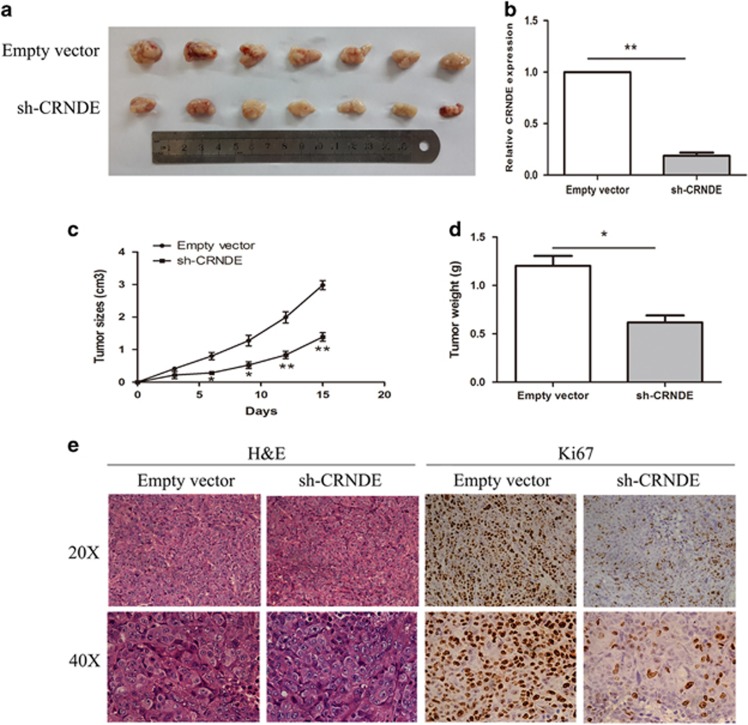
Knockdown of CRNDE inhibits CRC tumorigenesis *in vivo.* (**a**) The total numbers of tumors after removal from the mice. (**b**) qRT-PCR analyses indicated that the CRNDE expression was significantly increased *in vivo*. (**c**) The tumor volumes were calculated every 3 days after inoculation. (**d**) The tumor weights after the tumors were harvested. The data represent the mean±S.D. (**e**) Representative images of H&E and immunohistochemical staining of the tumor. IHC revealed a downregulation of the proliferation index Ki-67. *P<0.05 and **P<0.01

To determine whether the level of CRNDE expression could affect CRC tumorigenesis *in vivo*, sh-CRNDE or negative control-transfected HCT116 cells were subcutaneously inoculated into male nude mice. At 15 days after injection, the tumors formed in the sh-CRNDE group were dramatically smaller than that in the negative control group (*P*<0.05) (Figure 4a). Correspondingly, the tumor volumes and weights were obviously decreased compared with the controls (*P*<0.05) (Figures 4b and c). Moreover, the qRT-PCR analysis confirmed that the expression level of CRNDE remained markedly reduced in the sh-CRNDE group (*P*<0.05) (Figure 4d). The immunohistochemistry (IHC) assay results shown that the tumors developed from sh-CRNDE cells displayed karyopyknosis, alterations in shape, and reduced Ki-67 staining compared with tumors formed from empty vector-transfected cells (Figures 4e). These results indicate that CRNDE is significantly associated with CRC cell proliferation *in vivo*.

### CRNDE knockdown increases the expression of genes involved in cell proliferation

**Figure 5:**
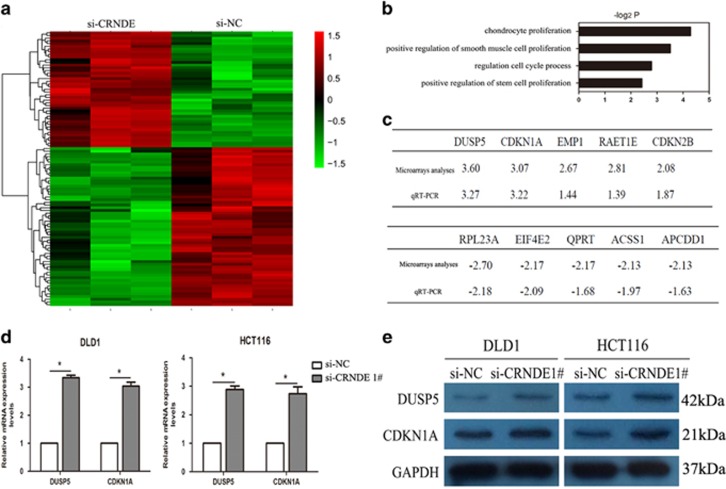
CRNDE knockdown increases the expression of genes involved in cell proliferation. (**a**) Mean-centered, hierarchical clustering of transcripts altered in scrambled siRNA-treated cells and si-CRNDE-treated cells, with three repeats. (**b**) Gene Ontology analysis for all genes with altered expressions between si-NC- and si-CRNDE-treated DLD1 cells *in vitro*. (**c**) GSEA analysis indicated that cell proliferation process gene sets were significantly altered in genes upregulated by CRNDE knockdown. qRT-PCR was used to validate the changes of several mRNAs involved in cell proliferation. (**d**) qRT-PCR was used to validate the changes of DUSP5 and CDKN1A mRNAs after si-CRNDE 1 or si-NC transfection in DLD1 and HCT116 cells. (**e**) Western blot analysis of DUSP5 and CDKN1A after si-CRNDE 1 or si-NC transfection in DLD1 and HCT116 cells. GAPDH protein was used as an internal control. *P<0.05

To obtain unbiased findings on the CRNDE- associated pathway, we assessed the gene expression profiles of CRC cells in which CRNDE expression was suppressed. Specifically, we performed RNA transcriptome sequencing from control and CRNDE-depleted DLD1 cells. DLD1 cells were treated with si-NC or si-CRNDE 1 for 48 h. To further study the involved pathways activated by CRNDE, we analyzed the associated genes using data collected from the Gene Ontology (GO) database (Figure 5a). The most prominent GO categories were related to cell proliferation, suggesting that these biological processes are particularly affected in CRNDE-knockdown cells (Figure 5b). The GO results were essentially in agreement with our experimental findings. The qRT-PCR was then used to confirm the changes in the levels of several upregulated or downregulated mRNAs involved in cell proliferation. The results showed that knockdown of CRNDE increased DUSP5, CDKN1A, EMP1, RAET1E, and CDKN2B expression, but decreased RPL23A, EIF4E2,QPRT, ACSS1, and APCDD1 expression (Figure 5c). To observe the key downstream mediators of CRNDE in CRC cells, we chose the DUAP5 and CDKN1A mRNAs for further studies because they exhibited the highest expression levels in CRNDE-knockdown cells. The qRT-PCR analysis confirmed that the expression level of DUAP5 and CDKN1A remained markedly increased in the si-CRNDE group (*P*<0.05) (Figure 5d). In addition, western blot analysis demonstrated that DUAP5 and CDKN1A proteins were increased in si-CRNDE-treated CRC cells (*P*<0.05) (Figure 5e). These findings indicate that the dysregulated genes may be key downstream mediators of CRNDE.

### CRNDE epigenetically silences DUSP5 and CDNK1A transcription by binding with EZH2

**Figure 6:**
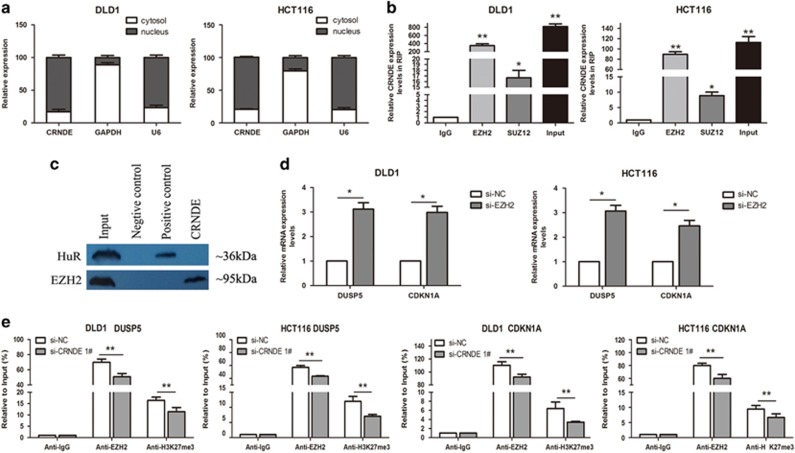
CRNDE epigenetically silences DUSP5 and CDKN1A transcription by binding to EZH2. (**a**) qRT-PCR analysis of CRNDE nuclear and cytoplasmic expression levels in DLD1 and HCT116 cells. U6 was used as a nucleus marker, and GAPDH was used as a cytosol marker. (**b**) RIP experiments were performed in DLD1 and HCT116 cells, and the coprecipitated RNA was subjected to qRT-PCR for CRNDE. The fold enrichment of CRNDE in EZH2/SUZ12 RIP is relative to its matched IgG control. (**c**) The RNA pull-down experiments also confirmed CRNDE could interact with EZH2 in DLD1 cell line. Protein levels in immunoprecipitates were determined by western blot assay. The expression levels of EZH2 protein were presented. (**d**) qRT-PCR was used to validate the changes of DUSP5 and CDKN1A mRNAs after si-EZH2 or si-NC transfection in DLD1 and HCT116 cells. (**e**) ChIP-qRT-PCR of EZH2 occupancy and H3K27me3 binding in the DUSP5 and CDKN1A promoters in DLD1 and HCT116 cells treated with si-CRNDE 1 (48 h) or si-NC; IgG as a negative control. Error bars indicate mean±S.D. *P<0.05 and **P<0.01

Although CRNDE exhibits vital biological functions in CRC, the underlying molecular mechanisms involved in its interactions are complex and diverse. CRNDE is previously found to be overexpressed in CRC and promotes its cell proliferation via acting as endogenous competing RNAs (ceRNAs) for miRNAs. However, its other molecular mechanism and downstream targets involved in regulation of CRC cell phenotype are not known. To investigate the potential mechanism and downstream targets of CRNDE in CRC cells, we first analyzed its distribution in CRC cells and found that CRNDE is mostly located in nucleus (*P*<0.05) (Figure 6a), implying that CRNDE may be involved in transcriptional regulation.

Then, we predicted the possible binding proteins of CRNDE by literature and found that it could bind to histone modification enzymes. Recently, several studies have concluded that ∼20% of lncRNAs can regulate downstream target genes by binding with PRC2. PRC2 is a methyltransferase that trimethylates H3K27 to suppress the transcription of specific genes. The RIP assay was performed to further investigate whether CRNDE could bind to PRC2. The results showed that CRNDE could interact with EZH2 and SUZ12 (suppressor of zeste 12 homolog) (the two key components of PRC2) in DLD1 and HCT116 cells (Figure 6b). The RNA pull-down experiments also confirmed CRNDE could interact with EZH2 in DLD1 cell line (Figure 6c). Our findings indicate that CRNDE may epigenetically inhibit downstream target genes by binding to EZH2.

Furthermore, we found that the expression levels of DUSP5 and CDKN1A were increased in si-EZH2-treated CRC cells (*P*<0.05) (Figure 6d), implying DUSP5 and CDKN1A may be the key downstream genes of CRNDE, and CRNDE can inhibit its expression by binding to EZH2. The results of chromatin immunoprecipitation (ChIP) assays showed that EZH2 could directly bind to DUSP5 and CDKN1A promoter regions and induce the histone H3 lysine 27 trimethylation (H3K27me3) modification in DLD1 and HCT116 cells. Knockdown of CRNDE resulted in reduced EZH2 binding and H3K27me3 occupancy of the DUSP5 and CDKN1A promoter (*P*<0.01) (Figure 6e). These results suggest that CRNDE could promote CRC cell growth partly through epigenetically silencing DUSP5 and CDKN1A transcription by binding to EZH2.

### DUSP5 functions as the tumor suppressor in CRC

**Figure 7:**
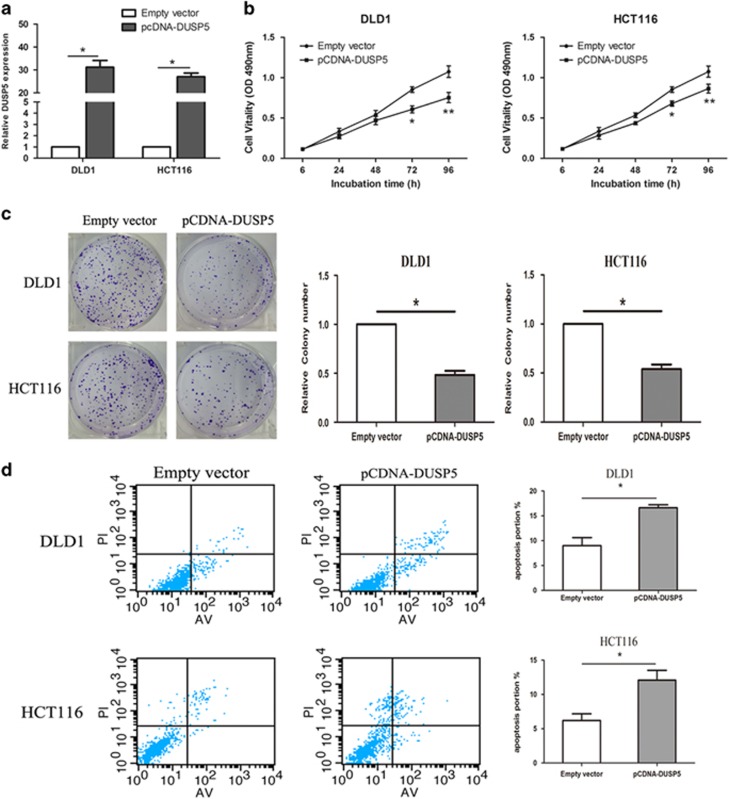
DUSP5 function as the tumor suppressor in CRC. (**a**) qRT-PCR analysis of DUSP5expression levels following the treatment of DLD1 and HCT116 cells with pcDNA- DUSP5 or empty vector. (**b**) A MTT assay was performed to determine DLD1 and HCT116 cell proliferation following treatment with pcDNA- DUSP5 or empty vector. The data represent the mean±S.D. from three independent experiments. (**c**) Colony-forming growth assays were performed to determine CRC cell proliferation. The colonies were counted and captured. (**d**) The percentage of apoptotic cells was determined by flow cytometric analysis. The data represent the mean±S.D. from three independent experiments. *P<0.05 and **P<0.01

To further confirm the biological function of DUSP5 in CRC, the overexpression plasmid pcDNA-DUSP5 was transfected into DLD1and HCT116 cells that effectively enhanced the expression of DUSP5 (*P*<0.05) (Figure 7a). We performed MTT and colony formation assays to determine cell viability. The results revealed that the cell vitality of DLD1 and HCT116 cells transfected with pcDNA-DUSP5 was significantly inhibited compared with empty vector-treated groups (*P*<0.05) (Figures 7b and c). Then, the flow cytometric analysis demonstrated that the upregulation of DUSP5 expression significantly increased in CRC cell apoptosis (*P*<0.05, Figure 7d). These data suggest that DUSP5 inhibited the proliferation and promoted apoptosis of CRC cells.

### DUSP5 silencing potentially involves the oncogenic function of CRNDE

**Figure 8:**
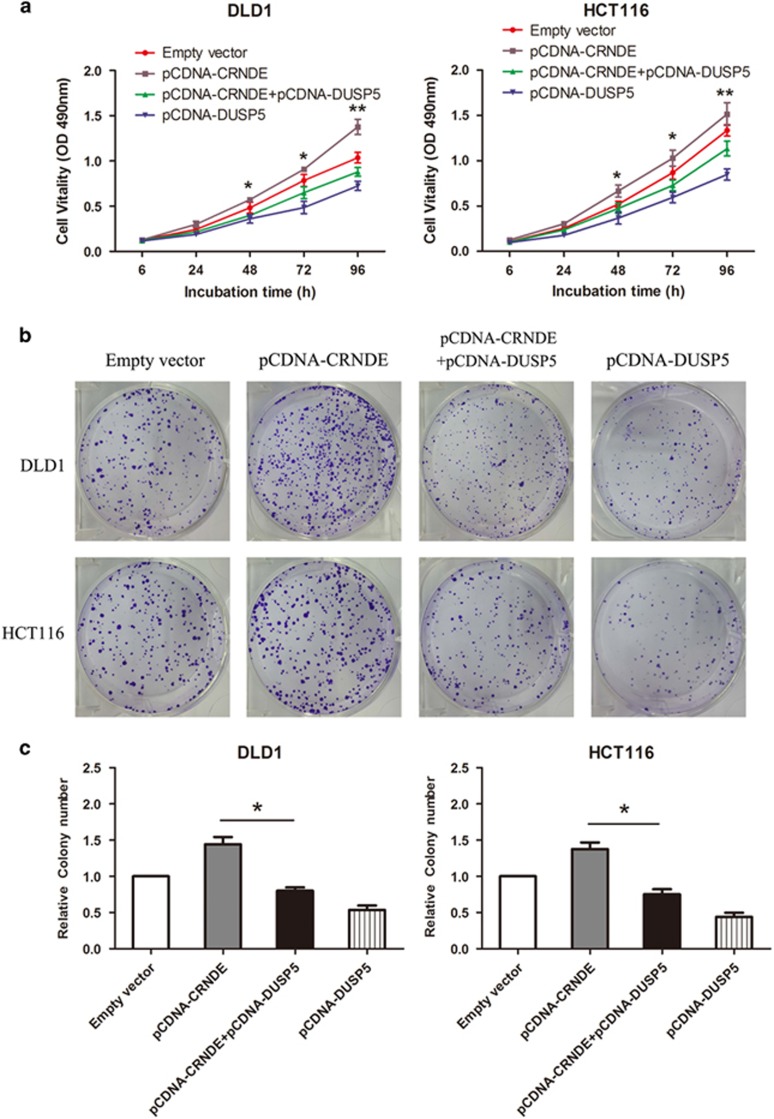
Silencing DUSP5 potentially involves the oncogenic function of CRNDE. (**a**) MTT assays were used to determine the cell viability for pcDNA-CRNDE and pcDNA-DUSP5 co-transfected DLD1 and HCT116 cells. (**b**) Colony-forming growth assays were performed to determine CRC cell proliferation. (**c**) The colonies were counted and captured. Experiments were performed in triplicate. Error bars indicate mean±S.D. *P<0.05 and **P<0.01

To validate whether CRNDE regulates CRC cell proliferation by silencing DUSP5 expression, rescue assays were performed. DLD1 and HCT116 cells were co-transfected with pcDNA-DUSP5 and pcDNA-CRNDE, and the MTT and colony formation assay results indicated that pcDNA-DUSP5 and pcDNA-CRNDE co-transfection partially rescues pcDNA-CRNDE-promoted proliferation ability (*P*<0.05) (Figures 8a–c). These results indicated that the effect of CRNDE on CRC partially involves targeting DUSP5.

## Discussion

LncRNAs are broadly defined as endogenous cellular RNAs molecules longer than 200 nt in length with limited or no protein-coding capacity and capable of regulating gene expression^26^ at various levels, including chromatin modification, transcription, and post-transcriptional processing.^9, 27, 28^ Recently, more evidence has emerged that dysregulation of lncRNAs may be an important class of pervasive genes and the future diagnostic, therapeutic, or prognostic cancer biomarkers involved in carcinogenesis and development.^13, 29^ For instance, our previous studies showed that lncRNA SPRY4-IT1 (SPRY4 intronic transcript 1) could be a growth regulator, and promoted the proliferation of estrogen receptor (−) human breast cancer cells by upregulating the expression of zinc-finger protein 703;^30^ lncRNA HOXA-AS2 promotes gastric cancer cells proliferation by epigenetically silencing CDKN1A, PLK3, and DDIT3 expression;^17^ lncRNA FOXP4-AS1 (FOXP4 antisense RNA 1) is an unfavorable prognostic factor and regulates proliferation and apoptosis in colorectal cancer.^31^

LncRNA CRNDE was long considered to an oncogene in the occurrence and development of CRC, indicating a poor prognosis.^20, 23^ The underlying molecular mechanisms involved in CRNDE interactions are complex and diverse. Recent studies have revealed the multilayered biological function of CRNDE cell proliferation, metastasis, and chemoresistance by regulating miRNAs.^24, 25^ Against this background, there is an urgent need to identify CRNDE and investigate its more effective and complex mechanisms. Here, we revealed a novel mechanism and thoroughfare of CRNDE in CRC cells.

In this study, we found that the expression of CRNDE in CRC tissues was highest by the network data, suggesting that CRNDE plays a crucial role in CRC and may be an independent clinical marker in CRC prognosis and therapy. Then, we identified this result by tissue validation and found that CRNDE overexpression was positively correlated with advanced pathological stages and larger tumor sizes. In addition, the knockdown of CRNDE significantly suppressed proliferation of CRC cells both *in vitro* and *in vivo* by causing G1 arrest and promoting apoptosis.

We analyzed the downstream target genes of CRNDE by microarray analysis, and the data showed that DUSP5 and CDKN1A might be the downstream mediators via affecting the cell proliferation pathways. DUSP5 is a negative regulator of MAPK signaling pathway and has recently been identified as a tumor suppressor in several human malignancies, including CRC.^32, 33, 34, 35^ Recent studies found that the importance of feedback mechanism is illustrated by the fact that expression of oncogenic BRAFV600E, a feedback-insensitive mutant RAF kinase, reprograms DUSP5 into a cell-wide ERK inhibitor that facilitates cell proliferation and transformation.^36^ CDKN1A (p21), one of the CDK inhibitor, is expressed ubiquitously and plays multifarious roles in many cellular processes.^37^ Our previous studies have also shown that CDKN1A is a critical molecule for inhibiting cell proliferation in CRC cells.^17^ Here, we also demonstrate that DUSP5 can act as a tumor suppressor and is silenced by CRNDE in CRC cells. Meanwhile, CRNDE can also suppress the expression of CDKN1A.

Lots of evidence suggested that the importance of lncRNAs in human cancer may be associated with their abilities to impact cellular functions through regulating target gene expression through binding to various histone modification enzymes.^29, 38, 39^ PRC2, which consists of EZH2, SUZ12, and EED (embryonic ectoderm development), is a typical methyltransferase for histone H3K27me3.^40^ For example, lncRNA HOXA11-AS could promote proliferation and invasion of gastric cancer by scaffolding the chromatin modification factors PRC2, LSD1, and DNMT1;^29^ long intergenic noncoding RNA 00511 acts as an oncogene in non-small-cell lung cancer by binding to EZH2 and suppressing p57.^41^

To further grope for the potential molecular mechanisms involved, we performed the nucleocytoplasmic separation experiments and found that CRNDE RNA is mainly distributed in the nucleus, indicating that CRNDE may exert transcriptional regulation function. The RIP and RNA pull-down assays revealed that CRNDE could directly bind with EZH2 in DLD1 and HCT116 cells to silence DUSP5 and CDKN1A expression. The results of ChIP analysis demonstrated that EZH2 could directly bind to DUSP5 and CDKN1A promoter regions and induce H3K27me3 modification in CRC cells. These results revealed that CRNDE promotes CRC cell proliferation is a manner that is dependent on the regulation of DUSP5 and CDKN1A expression by binding to EZH2.

In conclusion, our study provides the evidence that CRNDE was upregulated in CRC tissues and its high expression level may be associated with CRC patients’ progression of disease. The promotion role of CRNDE on CRC cell proliferation and tumorigenesis may partly via epigenetically restraining DUSP5 and CDKN1A transcription by binding with EZH2 and inhibiting EZH2-mediated methylation modification of these target genes. CRNDE may serve as a potential target for diagnosis and treatment in human CRC.

## Materials and methods

### Differential expression analysis

CRC gene expression data were downloaded from the GEO data set. The independent data set from GSE21510 (http://www.ncbi.nlm.nih.gov/geo/query/acc.cgi?acc=GSE21510) was included in this study and normalized using the Robust Multichip Average. After we had downloaded probe sequences from GEO or the microarray manufacturers, Blast+2.2.30 was used to reannotate the probes in the GENCODE Release 20 sequence databases (http://www.gencodegenes.org/) for lncRNA.

### Tissue collection and ethics statement

A total of 80 paired tumor tissues and matched normal tissues were collected from CRC patients who received surgical treatment between January 2011 and December 2014 at Second Affiliated Hospital of Nanjing Medical (China). None of the patients received any local or systemic treatment before surgery. The pathological stage, grade, and nodal status were appraised by an experienced pathologist. All experiments were approved by the research ethics committee of Nanjing Medical University, China. Written informed consent was obtained from all patients. The animal experiments were performed with the approval of the institutional committee for animal research and conformed to national guidelines for the care and use of laboratory animals.

### Cell lines and culture conditions

The human CRC cell lines (DLD1, HCT116, SW480, and LOVO cells) and the human colonic epithelial cells HCoEpiC were purchased from American Type Culture Collection (Manassas, VA, USA). The cells were cultured in Dulbecco’s modified Eagle’s medium (DMEM), (Invitrogen, Grand Island, NY, USA) in humidified air at 37 °C with 5% CO_2_. All media were supplemented with 10% fetal bovine serum (FBS), 100 U/ml penicillin, and 100 mg/ml streptomycin (Invitrogen).

### RNA extraction and qRT-PCR analyses

Total RNA was extracted from tissues or cultured cells using TRIZOL reagent (Invitrogen). For qRT-PCR, RNA was reverse transcribed to cDNA using a Reverse Transcription Kit (Takara, Dalian, China). Real-time PCR analyses were performed with SYBR Premix Ex Taq (Takara). Results were normalized to the expression of GAPDH. The qRT-PCR assays were conducted on the ABI 7500 (ABI, Foster, CA, USA) and data collected with this instrument. Our qRT-PCR results were analyzed and expressed relative to threshold cycle (CT) values, and then converted to fold changes. Each sample was analyzed in triplicate. The primer sequences used for the studies are presented in Supplementary Table S1.

### Transfection of CRC cells

Two individual CRNDE (CRNDE 1 and 2), EZH2, and scrambled negative control (NC) siRNAs were purchased from Invitrogen and transfected into cells using Lipofectamine 2000 (Invitrogen, USA). Plasmid vectors (sh-CRNDE, sh-DUSP5, and empty vector) for transfection were extracted by DNA Midiprep kit (Qiagen, Hilden, Germany) and transfected into cells using Fugene (Roche, Basel, Switzerland). The sequences of the siRNAs are described in Supplementary Table S2. The full-length complementary DNA of CRNDE was synthesized by Realgene (Nanjing, China) and subcloned into the pcDNA3.1 (+) vector (Invitrogen) according to the manufacturer’s instructions. Cells were harvested after 48 h for qRT-PCR and western blot analyses.

### Cell proliferation assays

Cell viability was tested with a Cell Proliferation Reagent Kit I (MTT) (Roche). At 48 h after si-CRNDE 1, 2, or pCDNA-CRNDE transfection with a negative control vector, 3000 cells per well were allowed to grow in 96-well plates with 5 replicate wells. After 6 h of culture as well as at 24, 48, 72, and 96 h after starting the culture, the cells were treated with 100 *μ*g MTT by adding it to the medium. The cells were incubated at 37 °C for an additional 4 h. Then, the medium was removed, and dimethylsulfoxide (DMSO) was added for 10 min to lyse the cells. Finally, the absorbance was measured at 490 nm. All experiments were performed in triplicate.

### Colony formation and clonogenic assays

Cells were trypsinized into single-cell suspensions 48 h after transfection. For the colony formation assay, 1000 transfected cells were placed in each well of 6-well plates and maintained in appropriate medium containing 10% FBS for 2 weeks and the medium was replaced every 4 days. After 14 days, the colonies were fixed with methanol and stained with 0.1% crystal violet (Sigma, St. Louis, MO, USA) in PBS for 15 min. The visible colonies were manually counted. For each treatment group, wells were assessed in triplicate, and experiments were independently repeated three times.

### Flow cytometry

DLD1 and HCT116 cells transfected with si-CRNDE 1, 2, or pCDNA-CRNDE were harvested 48 h after transfection by trypsinization. After double staining with FITC-Annexin V and propidium iodide (PI) had been performed using the FITC Annexin V Apoptosis Detection Kit (BD Biosciences), in accordance with the manufacturer’s recommendations, the cells were analyzed by flow cytometry (FACScan; BD Biosicences, Franklin Lakes, NJ, USA) with CellQuest software (BD Biosicences). Cells were classified into viable cells, dead cells, early apoptotic cells, and apoptotic cells, and then the relative ratio of early apoptotic cells was compared with that of the control transfectant for each experiment. All samples were assayed in triplicate.

### Western blot analysis and antibodies

Cell protein lysates were separated by 10% sodium dodecyl sulfate polyacrylamide gel electrophoresis (SDS-PAGE), transferred to 0.22 *μ*m NC membranes (Sigma), and incubated with specific antibodies. ECL chromogenic substrate was used for quantification by densitometry (Quantity One software; Bio-Rad, Hercules, CA, USA). A GAPDH (2118, Cell Signaling Technology, Inc. (CST, Boston, MA, USA)), USA) antibody was used as a control. The anti-CDK2 (2546, CST), anti-CDK6 (13331, CST), and CDKN1A (3698, CST) (all 1 : 1000) antibodies were purchased from CST. The anti-DUSP5 (ab54939, Abcam, MA, USA) (1 : 1000) antibody was purchased from Sigma.

### Tumor formation assay in a nude mouse model

Male athymic BALB/c nude mice (4 weeks old) were maintained under pathogen-free conditions and manipulated in accordance with the protocols approved by the Shanghai Medical Experimental Animal Care Commission. HCT116 cells were stably transfected with sh-CRNDE or empty vector, harvested from 6-well cell culture plates, washed with PBS, and resuspended at a concentration of 1 × 108 cells/ml. A total of 100 *μ*l of suspended cells was subcutaneously injected into a single side of the posterior flank of each mouse. Tumor growth was examined every 3 days, and tumor volumes were calculated using the following formula: 0.5 × length × width^2^. At 2 weeks after cell injection, the mice were killed. The subcutaneous weight of each tumor was measured and the tumors were used for further analysis. The protocol was approved by the committee on the ethics of animal experiments of the Nanjing Medical University.

### IHC analysis

Tumor tissue samples were immunostained for H&E and Ki-67 as described previously.^17^ The expression was considered to be positive when ⩾50% cancer cells were stained.

### Microarray data analysis

RNA extraction and purification, RNA amplification and labeling, array hybridization, and data acquisition were performed as described in the Affymetrix (Santa Clara, CA, USA) manufacturer’s instructions. Affymetrix Human U133 Plus 2.0 whole genome chips were used. Array hybridization and wash was performed using GeneChip Hybridization, Wash and Stain Kit (cat. no. 900720, Affymetrix) in Hybridization Oven 645 (cat. no. 00-0331-220V, Affymetrix) and Fluidics Station 450 (cat. no. 00-0079, Affymetrix) following the manufacturer’s instructions. Slides were scanned by GeneChip Scanner 3000 (cat. no. 00-00212, Affymetrix) and Command Console Software 4.0 (Affymetrix) with default settings. Raw data were normalized by MAS 5.0 algorithm, Gene Spring Software 11.0 (Agilent Technologies, Santa Clara, CA, USA).

### Subcellular fractionation location

The separation of nuclear and cytosolic fractions was performed using the PARIS Kit (Life Technologies, Carlsbad, CA, USA) according to the manufacturer’s instructions.

### RNA immunoprecipitation (RIP) assay

RIP experiments were performed using the Magna RIP RNA-Binding Protein Immunoprecipitation Kit (Millipore, Billerica, MA, USA) following the manufacturer’s instructions. Antibody for RIP assays of EZH2, SUZ12, or control IgG were from Millipore.

### RNA pull-down assays

CRNDE RNAs were *in vitro* transcribed using T7 RNA polymerase (Ambio Life, Austen, TX, USA) that were then purified using the RNeasy Plus Mini Kit (Qiagen) and treated with RNase-free DNase I (Qiagen). Transcribed RNAs were biotin-labeled with the Biotin RNA Labeling Mix (Ambio Life). Positive, negative, and biotinylated RNAs were mixed and incubated with DLD1 cell lysates. Magnetic beads were added to each binding reaction, followed by incubation at room temperature. Then, the beads were washed with washing buffer. The eluted proteins were detected by western blot analysis.

### ChIP assay

CRC cells were treated with formaldehyde and incubated for 10 min to generate DNA–protein crosslinks. Cell lysates were then sonicated to generate chromatin fragments of 200 to 300 bp and immunoprecipitated with H3K27me3 and EZH2-specific antibody (CST) or IgG as control. Precipitated chromatin DNA was recovered and analyzed by qRT-PCR. The primer sequences used for the studies are presented in Supplementary Table S3.

### Statistical analysis

All experiments were repeated at least three times with each sample in triplicate. Sample sizes for relevant experiment were determined by power analysis. All data were expressed as mean±S.D. and analyzed using Student’s *t-*test to compare two groups of *in vitro* and *in vivo* data using the SPSS 17.0 software program (version 17.0, SPSS Inc, Armonk, NY, USA). A value of *P*<0.05 was considered to be statistically significant.

## Figures and Tables

**Table 1:** Correlation between CRNDE expression and clinicopathological characteristics of CRC patients

**Characteristics**	**Number (*****n*****=80)**	**Percent**	**CRNDE**		***P***, ***χ***^**2**^ **test** ***P*****-value**
			**High**	**Low**	
*Age (years)*					0.370
<60	43	53.75%	24	19	
⩾60	37	46.25%	16	21	
					
*Gender*					0.367
Male	45	56.25%	25	20	
Female	35	43.75%	15	20	
					
*Maximum tumor size*					0.003^a^
<5 cm	44	55%	15	29	
⩾5 cm	36	45%	25	11	
					
*Location*					0.650
Colon	33	41.25%	18	15	
Rectum	47	58.75%	22	25	
					
*Depth of tumor*					0.254
T1 and T2	48	60%	27	21	
T3 and T4	32	40%	13	19	
					
*Tumor stage*					0.001^a^
I and II	49	61.25%	17	32	
III and IV	31	38.75%	23	8	

a *P*<0.05 was considered significant (*χ*^2^ test between two groups)

## References

[bib1] Siegel RL, Miller KD, Jemal A. Cancer statistics, 2016. CA Cancer J Clin 2016; 66: 7–30. 26742998 10.3322/caac.21332

[bib2] Siegel RL, Miller KD, Jemal A. Cancer Statistics, 2017. CA Cancer J Clin 2017; 67: 7–30. 28055103 10.3322/caac.21387

[bib3] Chen W, Zheng R, Baade PD, Zhang S, Zeng H, Bray F et al. Cancer statistics in China, 2015. CA Cancer J Clin 2016; 66: 115–132. 26808342 10.3322/caac.21338

[bib4] Edwards BK, Noone AM, Mariotto AB, Simard EP, Boscoe FP, Henley SJ et al. Annual Report to the Nation on the status of cancer, 1975-2010, featuring prevalence of comorbidity and impact on survival among persons with lung, colorectal, breast, or prostate cancer. Cancer 2014; 120: 1290–1314. 24343171 10.1002/cncr.28509 PMC3999205

[bib5] Colussi D, Brandi G, Bazzoli F, Ricciardiello L. Molecular pathways involved in colorectal cancer: implications for disease behavior and prevention. Int J Mol Sci 2013; 14: 16365–16385. 23965959 10.3390/ijms140816365 PMC3759916

[bib6] Ponting CP, Oliver PL, Reik W. Evolution and functions of long noncoding RNAs. Cell 2009; 136: 629–641. 19239885 10.1016/j.cell.2009.02.006

[bib7] Esteller M. Non-coding RNAs in human disease. Nat Rev Genet 2011; 12: 861–874. 22094949 10.1038/nrg3074

[bib8] Xu Z, Yan Y, Qian L, Gong Z. Long non-coding RNAs act as regulators of cell autophagy in diseases (Review). Oncol Rep 2017; 37: 1359–1366. 28184916 10.3892/or.2017.5416 PMC5364869

[bib9] Kung JT, Colognori D, Lee JT. Long noncoding RNAs: past, present, and future. Genetics 2013; 193: 651–669. 23463798 10.1534/genetics.112.146704 PMC3583990

[bib10] Li CQ, Huang GW, Wu ZY, Xu YJ, Li XC, Xue YJ et al. Integrative analyses of transcriptome sequencing identify novel functional lncRNAs in esophageal squamous cell carcinoma. Oncogenesis 2017; 6: e297. 28194033 10.1038/oncsis.2017.1 PMC5337622

[bib11] Wu Y, Wang YQ, Weng WW, Zhang QY, Yang XQ, Gan HL et al. A serum-circulating long noncoding RNA signature can discriminate between patients with clear cell renal cell carcinoma and healthy controls. Oncogenesis 2016; 5: e192. 26878386 10.1038/oncsis.2015.48 PMC5154346

[bib12] Yin DD, Liu ZJ, Zhang E, Kong R, Zhang ZH, Guo RH. Decreased expression of long noncoding RNA MEG3 affects cell proliferation and predicts a poor prognosis in patients with colorectal cancer. Tumour Biol 2015; 36: 4851–4859. 25636452 10.1007/s13277-015-3139-2

[bib13] Shi Y, Liu Y, Wang J, Jie D, Yun T, Li W et al. Downregulated long noncoding RNA BANCR promotes the proliferation of colorectal cancer cells via downregualtion of p21 expression. PLoS ONE 2015; 10: e0122679. 25928067 10.1371/journal.pone.0122679 PMC4415816

[bib14] Lian Y, Wang J, Feng J, Ding J, Ma Z, Li J et al. Long non-coding RNA IRAIN suppresses apoptosis and promotes proliferation by binding to LSD1 and EZH2 in pancreatic cancer. Tumour Biol 2016; 37: 14929–14937. 27644252 10.1007/s13277-016-5380-8

[bib15] Zhang YH, Fu J, Zhang ZJ, Ge CC, Yi Y. LncRNA-LINC00152 down-regulated by miR-376c-3p restricts viability and promotes apoptosis of colorectal cancer cells. Am J Transl Res 2016; 8: 5286–5297. 28078002 PMC5209482

[bib16] Xiong DD, Feng ZB, Cen WL, Zeng JJ, Liang L, Tang RX et al. The clinical value of lncRNA NEAT1 in digestive system malignancies: a comprehensive investigation based on 57 microarray and RNA-seq datasets. Oncotarget 2017; 8: 17665–17683. 28118609 10.18632/oncotarget.14756 PMC5392277

[bib17] Ding J, Xie M, Lian Y, Zhu Y, Peng P, Wang J et al. Long noncoding RNA HOXA-AS2 represses P21 and KLF2 expression transcription by binding with EZH2, LSD1 in colorectal cancer. Oncogenesis 2017; 6: e288. 28112720 10.1038/oncsis.2016.84 PMC5294247

[bib18] Ding J, Lu B, Wang J, Wang J, Shi Y, Lian Y et al. Long non-coding RNA Loc554202 induces apoptosis in colorectal cancer cells via the caspase cleavage cascades. J Exp Clin Cancer Res 2015; 34: 100. 26362196 10.1186/s13046-015-0217-7 PMC4567799

[bib19] Lian Y, Ding J, Zhang Z, Shi Y, Zhu Y, Li J et al. The long noncoding RNA HOXA transcript at the distal tip promotes colorectal cancer growth partially via silencing of p21 expression. Tumour Biol 2016; 37: 7431–7440. 26678886 10.1007/s13277-015-4617-2

[bib20] Graham LD, Pedersen SK, Brown GS, Ho T, Kassir Z, Moynihan AT et al. Colorectal neoplasia differentially expressed (CRNDE), a novel gene with elevated expression in colorectal adenomas and adenocarcinomas. Genes Cancer 2011; 2: 829–840. 22393467 10.1177/1947601911431081 PMC3278902

[bib21] Zheng J, Liu X, Wang P, Xue Y, Ma J, Qu C et al. CRNDE promotes malignant progression of glioma by attenuating miR-384/PIWIL4/STAT3 axis. Mol Ther 2016; 24: 1199–1215. 27058823 10.1038/mt.2016.71 PMC5088760

[bib22] Dong R, Liu XQ, Zhang BB, Liu BH, Zheng S, Dong KR. Long non-coding RNA-CRNDE: a novel regulator of tumor growth and angiogenesis in hepatoblastoma. Oncotarget 2017; 8: 42087–42097. 28178668 10.18632/oncotarget.14992 PMC5522051

[bib23] Liu T, Zhang X, Gao S, Jing F, Yang Y, Du L et al. Exosomal long noncoding RNA CRNDE-h as a novel serum-based biomarker for diagnosis and prognosis of colorectal cancer. Oncotarget 2016; 7: 85551–85563. 27888803 10.18632/oncotarget.13465 PMC5356757

[bib24] Han P, Li JW, Zhang BM, Lv JC, Li YM, Gu XY et al. The lncRNA CRNDE promotes colorectal cancer cell proliferation and chemoresistance via miR-181a-5p-mediated regulation of Wnt/beta-catenin signaling. Mol Cancer 2017; 16: 9. 28086904 10.1186/s12943-017-0583-1 PMC5237133

[bib25] Gao H, Song X, Kang T, Yan B, Feng L, Gao L et al. Long noncoding RNA CRNDE functions as a competing endogenous RNA to promote metastasis and oxaliplatin resistance by sponging miR-136 in colorectal cancer. Onco Targets Ther 2017; 10: 205–216. 28115855 10.2147/OTT.S116178 PMC5221653

[bib26] Nagano T, Fraser P. No-nonsense functions for long noncoding RNAs. Cell 2011; 145: 178–181. 21496640 10.1016/j.cell.2011.03.014

[bib27] Gibb EA, Brown CJ, Lam WL. The functional role of long non-coding RNA in human carcinomas. Mol Cancer 2011; 10: 38. 21489289 10.1186/1476-4598-10-38 PMC3098824

[bib28] Ji Q, Zhang L, Liu X, Zhou L, Wang W, Han Z et al. Long non-coding RNA MALAT1 promotes tumour growth and metastasis in colorectal cancer through binding to SFPQ and releasing oncogene PTBP2 from SFPQ/PTBP2 complex. Br J Cancer 2014; 111: 736–748. 25025966 10.1038/bjc.2014.383 PMC4134507

[bib29] Sun M, Nie F, Wang Y, Zhang Z, Hou J, He D et al. LncRNA HOXA11-AS promotes proliferation and invasion of gastric cancer by scaffolding the chromatin modification factors PRC2, LSD1, and DNMT1. Cancer Res 2016; 76: 6299–6310. 27651312 10.1158/0008-5472.CAN-16-0356

[bib30] Shi Y, Li J, Liu Y, Ding J, Fan Y, Tian Y et al. The long noncoding RNA SPRY4-IT1 increases the proliferation of human breast cancer cells by upregulating ZNF703 expression. Mol Cancer 2015; 14: 51. 25742952 10.1186/s12943-015-0318-0 PMC4350857

[bib31] Li J, Lian Y, Yan C, Cai Z, Ding J, Ma Z et al. Long non-coding RNA FOXP4-AS1 is an unfavourable prognostic factor and regulates proliferation and apoptosis in colorectal cancer. Cell Prolif 2017; 50: 1. 10.1111/cpr.12312 PMC6529074 27790757

[bib32] Shin SH, Park SY, Kang GH. Down-regulation of dual-specificity phosphatase 5 in gastric cancer by promoter CpG island hypermethylation and its potential role in carcinogenesis. Am J Pathol 2013; 182: 1275–1285. 23402999 10.1016/j.ajpath.2013.01.004

[bib33] Cai C, Chen JY, Han ZD, He HC, Chen JH, Chen YR et al. Down-regulation of dual-specificity phosphatase 5 predicts poor prognosis of patients with prostate cancer. Int J Clin Exp Med 2015; 8: 4186–4194. 26064329 PMC4443163

[bib34] Rushworth LK, Kidger AM, Delavaine L, Stewart G, van Schelven S, Davidson J et al. Dual-specificity phosphatase 5 regulates nuclear ERK activity and suppresses skin cancer by inhibiting mutant Harvey-Ras (HRasQ61L)-driven SerpinB2 expression. Proc Natl Acad Sci USA 2014; 111: 18267–18272. 25489104 10.1073/pnas.1420159112 PMC4280588

[bib35] Yan X, Liu L, Li H, Huang L, Yin M, Pan C et al. Dual specificity phosphatase 5 is a novel prognostic indicator for patients with advanced colorectal cancer. Am J Cancer Res 2016; 6: 2323–2333. 27822421 PMC5088295

[bib36] Kidger AM, Rushworth LK, Stellzig J, Davidson J, Bryant CJ, Bayley C et al. Dual-specificity phosphatase 5 controls the localized inhibition, propagation, and transforming potential of ERK signaling. Proc Natl Acad Sci USA 2017; 114: E317–E326. 28053233 10.1073/pnas.1614684114 PMC5255582

[bib37] Gartel AL, Radhakrishnan SK. Lost in transcription: p21 repression, mechanisms, and consequences. Cancer Res 2005; 65: 3980–3985. 15899785 10.1158/0008-5472.CAN-04-3995

[bib38] Shen Y, Wang Z, Loo LW, Ni Y, Jia W, Fei P et al. LINC00472 expression is regulated by promoter methylation and associated with disease-free survival in patients with grade 2 breast cancer. Breast Cancer Res Treat 2015; 154: 473–482. 26564482 10.1007/s10549-015-3632-8 PMC4854534

[bib39] Khalil AM, Guttman M, Huarte M, Garber M, Raj A, Rivea MD et al. Many human large intergenic noncoding RNAs associate with chromatin-modifying complexes and affect gene expression. Proc Natl Acad Sci USA 2009; 106: 11667–11672. 19571010 10.1073/pnas.0904715106 PMC2704857

[bib40] Conway E, Healy E, Bracken AP. PRC2 mediated H3K27 methylations in cellular identity and cancer. Curr Opin Cell Biol 2015; 37: 42–48. 26497635 10.1016/j.ceb.2015.10.003

[bib41] Sun CC, Li SJ, Li G, Hua RX, Zhou XH, Li DJ. Long intergenic noncoding RNA 00511 acts as an oncogene in non-small-cell lung cancer by binding to EZH2 and suppressing p57. Mol Ther Nucleic Acids 2016; 5: e385. 27845772 10.1038/mtna.2016.94 PMC5155326

