# Supplementary figures and images for: Long noncoding RNA CRNDE promotes colorectal cancer cell proliferation via epigenetically silencing DUSP5/CDKN1A expression
Source: Cell Death Dis. 2017 Aug 10;8(8):e2997–. doi: 10.1038/cddis.2017.328 (PMC5596537; doi:10.1038/cddis.2017.328)

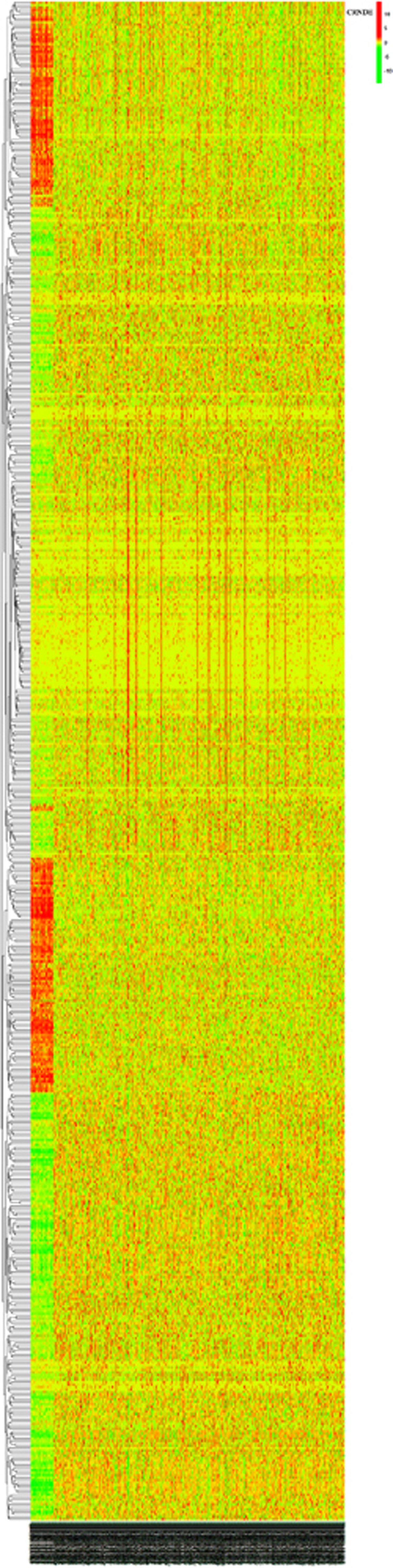

Supplement: Supplementary Information [file cddis2017328x4.tif]
