# Supplementary material for: Long noncoding RNA CRNDE promotes colorectal cancer cell proliferation via epigenetically silencing DUSP5/CDKN1A expression
Source: Cell Death Dis. 2017 Aug 10;8(8):e2997–. doi: 10.1038/cddis.2017.328 (PMC5596537; doi:10.1038/cddis.2017.328)
Supplement: Supplementary Information [file cddis2017328x5.doc]

CDDIS, Manuscript Number CDDIS-17-0273
Long non-coding RNA CRNDE promotes colorectal cancer cell proliferation via epigenetically silencing DUSP5 / CDKN1A expression

The legend/captions for supplementary Table 1-3 and Supplementary Figure 1:

1. **The captions for supplementary Figure 1:**

The microarray data downloaded from the TCGA and found that the CRNDE expression level was significantly up-regulated in CRC tissues compared with that in normal tissues.

1. **The captions for supplementary Table 1:**

The primer sequences of genes.

1. **The captions for supplementary Table 2:**

The primer sequences of si-RNAs.

1. **The captions for supplementary Table 3:**

The primer sequences used for the ChIP-qRT-PCR assay.
